# Influence of paternal education on maternal and child health outcomes in Sub-Saharan Africa: evidence from demographic and health surveys

**DOI:** 10.1186/s12978-025-02086-y

**Published:** 2025-07-29

**Authors:** Joshua Okyere, Demisu Zenbaba Hey, Sanni Yaya

**Affiliations:** 1 School of Human and Health Sciences, University of Huddersfield, Queensgate, Huddersfield, England, UK; 2 Department of Population and Health, University of Cape Coast, Cape Coast, Ghana; 3 School of Public Health, Madda Walabu University, Robe, Ethiopia; 4 The George Institute for Global Health, Imperial College, London, UK

**Keywords:** Paternal education, Maternal health, Child survival, Antenatal care, Skilled birth attendance, Sub-Saharan Africa, Demographic and health surveys

## Abstract

**Background:**

Maternal and child health outcomes remain pressing challenges in Sub-Saharan Africa (SSA), characterized by persistently high under-five mortality and inadequate utilization of essential maternal healthcare services. While the impact of maternal education on these outcomes is well documented, the influence of paternal education remains underexplored. This study investigates the association between paternal education and maternal health service utilization, focusing on antenatal care (ANC) and skilled birth attendance (SBA), as well as child survival across 22 SSA countries.

**Methods:**

We conducted a cross-sectional analysis using nationally representative data from Demographic and Health Surveys (DHS) administered between 2013 and 2024 in 22 SSA countries. The study sample included a weighted total of 109,818 children aged 0–59 months and their mothers. Key outcomes included maternal healthcare utilization (≥ 4 ANC visits and SBA) and child survival. Paternal education was classified into three categories: no education, primary education, and secondary or higher education. Logistic and ordered logistic regression models were employed, adjusting for maternal, child, household, and regional covariates.

**Results:**

Overall, 96.5% (95% CI: 96.4–96.6%) of children survived to age five, while 61.8% of mothers reported ≥ 4 ANC visits and 73.8% had skilled birth attendance. Higher paternal education was significantly associated with increased maternal healthcare utilization. Women whose partners had secondary or higher education were nearly twice as likely to attend ≥ 4 ANC visits (AOR: 1.99; 95% CI: 1.91–2.07) and more likely to access SBA (AOR: 1.60; 95% CI: 1.52–1.67) than those whose partners had no education. Marked regional and socioeconomic disparities persisted, with Southern SSA showing more favorable outcomes than Central and Eastern regions.

**Conclusion:**

Paternal education is strongly associated with improved maternal healthcare utilization, which is itself linked to enhanced child survival in SSA. Although no direct relationship was found between paternal education and child survival, these findings underscore the indirect but influential role fathers play in shaping health outcomes. Targeted strategies that promote male educational attainment and actively involve fathers in maternal and child health interventions—particularly in underserved regions—are essential for reducing health disparities and improving outcomes across the region.

## Background

Globally, substantial progress has been made in improving child health outcomes. Between 1990 and 2022, the global under-five mortality rate declined by 59%, from 93 to 37 deaths per 1,000 live births [1]. This remarkable reduction is largely attributed to strengthened health systems, expanded immunization programs, improved nutrition, and coordinated public health initiatives. However, despite these global gains, under-five and neonatal mortality remain disproportionately high in Sub-Saharan Africa (SSA), where progress has lagged behind other regions [2]. According to the World Health Organization (WHO), a child born in SSA is 11 times more likely to die within the first month of life than a child born elsewhere [1].

The persistently high mortality burden in SSA is driven by preventable and treatable conditions, including acute respiratory infections, diarrhea, and malaria, which continue to affect large segments of the population [3, 4]. In addition, maternal health behaviors significantly shape child survival outcomes. Inadequate antenatal care (ANC) attendance, home births, and the continued use of traditional birth attendants (TBAs) contribute to adverse outcomes for both mothers and infants [5, 6]. For instance, Amouzou et al. [6] reported that children delivered by skilled birth attendants were 16% less likely to die within the first 2–27 days of life compared to those delivered by TBAs, underscoring the critical importance of maternal healthcare services during the neonatal period.

Extant literature has identified several maternal factors that influence maternal and child health outcomes, ranging from demographic to behavioral determinants. Yaya et al. [7] highlighted maternal age, employment status, and place of residence as significant predictors of under-five mortality in SSA. Similarly, Tesfa et al. [8] demonstrated that shorter birth intervals and multiple births were associated with a heightened risk of under-five mortality. Socioeconomic challenges, such as poor maternal income levels, early age at first birth, and limited access to education, also contribute to poor health outcomes by impeding optimal ANC attendance and skilled birth delivery [9, 10].

One of the most consistent factors linked to improved maternal and child health outcomes is maternal education [7–9, 11, 12]. Education empowers women with knowledge about preventive healthcare, enhances their decision-making autonomy, and indirectly influences health outcomes through improved socioeconomic conditions [13, 14]. The Commission on Social Determinants of Health emphasized the critical role of education in shaping key determinants of health, such as income, employment opportunities, and living conditions [13]. As a result, the association between maternal education and maternal/child health outcomes is well-established in the literature.

In contrast, far less attention has been given to the influence of paternal education on maternal and child health outcomes. Although some studies have examined this area, their scope has often been limited to specific child health indicators such as developmental delays [15], child growth metrics [16], and perinatal outcomes like preterm birth and birth weight [17]. To the best of our knowledge, the only study which examined factors contributing to under-five mortality across 35 Sub-Saharan African countries, found paternal education to be statistically insignificant in explaining inequalities in under-five mortality, highlighting the dominant influence of maternal and socioeconomic factors [12].

Given the critical role fathers often play in decision-making, resource allocation, and family health behaviors in SSA, it is imperative to explore how paternal education may influence maternal and child health outcomes. This study investigates the role of paternal education as a key determinant of maternal healthcare utilization and child survival in sub-Saharan Africa. While maternal education has been widely recognized as a critical factor influencing health outcomes, less attention has been given to the impact of paternal education. However, few studies systematically examine paternal education as a determinant of maternal healthcare utilization and child survival across multiple Sub-Saharan African countries. This study fills a critical gap by offering a comprehensive, regionally diverse analysis of paternal influence, a dimension largely absent in existing maternal and child health literature. Understanding how paternal education shapes maternal healthcare behaviors and child survival outcomes is essential for designing comprehensive interventions that engage both parents in improving health outcomes.

## Methods

### Study setting and design

This study was conducted across 22 diverse countries in Sub-Saharan Africa (SSA), encompassing Angola, Benin, Burundi, Burkina Faso, Chad, Côte d’Ivoire, the Democratic Republic of Congo, Gabon, The Gambia, Ghana, Kenya, Lesotho, Madagascar, Mozambique, Namibia, Rwanda, Senegal, Sierra Leone, South Africa, Tanzania, Zambia, and Zimbabwe. These countries represent a wide range of socioeconomic and cultural contexts, providing a robust framework for understanding maternal and child health outcomes across the region.

### Study design and data source

The analysis utilized data from nationally representative Demographic and Health Surveys (DHS) conducted between 2013 and 2024 in the 22 selected countries. The DHS program collects comprehensive, standardized data on a variety of health, demographic, and socioeconomic indicators, including key exposures, confounding factors, and outcomes relevant to maternal and child health.

### Study population

The study population comprised children aged 0–59 months and their mothers. Inclusion criteria required complete data on child and maternal health outcomes, paternal education, and key sociodemographic variables. Participants with missing or implausible data for any outcome or exposure variables were excluded.

### Sample size and sampling methods

The DHS employs a two-stage cluster sampling design to ensure nationally and regionally representative data. In the first stage, clusters (enumeration areas) are selected within each country, followed by a second-stage random selection of households within each cluster. To improve representativeness, regions are stratified by urban and rural settings, and sample sizes are determined using a probability-proportional allocation method within each stratum. The dataset included a total of 109,818 children aged 0–59 months and their mothers, of whom 36,721 were from urban areas and 73,097 from rural areas.

### Data collection tool and quality assurance

Data were collected using three standardized instruments: the household questionnaire, the women’s questionnaire, and the men’s questionnaire. This study relied exclusively on data from the women’s questionnaire. Initially developed in English, the questionnaire was translated into major local languages specific to each country. Rigorous pretesting of survey instruments and comprehensive training sessions for data collectors, supervisors, and quality control personnel ensured high data quality and consistency.

### Study variable and measurements

This study explored the relationship between fathers’ education levels and health outcomes for mothers and their children in Sub-Saharan Africa. The health outcomes for mothers included whether they had skilled assistance during childbirth and the extent to which they utilized antenatal care (ANC) services. Skilled birth attendance was defined as having a qualified healthcare provider present during delivery, while ANC utilization was categorized based on the number of visits a mother made during pregnancy: no visits, 1–3 visits, or 4 or more visits. Child survival was assessed for all children under five years of age (*n* = 109,818). While ANC and SBA were analyzed in relation to the most recent birth, child survival analysis included all children under five to provide a broader assessment of mortality risk.

The key exposure variable for the study was fathers’ education levels, which were grouped into three categories: no formal education, primary education, and secondary or higher education. In addition to fathers’ education, the study considered several other factors that could influence maternal and child health. These included characteristics of the mother (such as her education level, age, media exposure, and her partner’s occupation), child-specific factors (such as their birth order and gender), household conditions (like wealth status and whether the household was in a rural or urban area), and regional differences across Eastern, Western, Central, and Southern Africa.

### Data extraction and analysis

The analysis was conducted using data from the Demographic and Health Surveys (DHS), which provide extensive and nationally representative information on health and demographics. The surveys follow a complex sampling process to ensure that results accurately represent the populations of each country. Using this data, the study employed statistical software (Stata, version 17) to conduct analyses.

First, descriptive statistics were generated to summarize the characteristics of the participants, including mothers, children, and their households. This involved calculating proportions and presenting them in tables and charts for clarity.

To explore the relationship between fathers’ education levels and the health outcomes of mothers and children, multilevel logistic regression models were used. This method allowed us to estimate how changes in fathers’ education were associated with the likelihood of positive outcomes, such as having skilled birth assistance. For the analysis of ANC utilization, a specialized model called multilevel ordered logistic regression was applied, as this outcome had multiple levels (no visits, 1–3 visits, or 4 + visits).

Initially, simple statistical tests were run to identify factors associated with health outcomes, including paternal education. Variables that appeared relevant (with a p-value below 0.2) were included in more comprehensive models to ensure that the findings accounted for other potential influencing factors. The final models provided adjusted odds ratios, which quantify the strength of the relationship between fathers’ education and health outcomes, while accounting for the effects of other variables.

Given the hierarchical nature of DHS data, we assessed the need for multilevel modeling by computing the Intraclass Correlation Coefficient (ICC) from an empty model. The ICC was found to be low, indicating minimal clustering effects. Accordingly, we used logistic regression models with robust standard errors to account for any potential clustering, ensuring valid inference.

Variables with a *p*-value of up to 0.2 in the multilevel bivariate regression analysis were considered for inclusion in the multilevel multivariable regression model. The variance inflation factor (VIF) was used to detect multicollinearity among individual-level determinants. The fixed effects of individual and community-level determinants on the outcome variables were reported using adjusted odds ratios (AOR) with 95% confidence intervals (CI). Statistical significance was determined at a *p*-value of ≤ 0.05.

### Ethical considerations and data set access

The study utilized publicly available anonymized datasets from the DHS program, covering surveys conducted between 2013 and 2024. The DHS complies with ethical standards, including obtaining informed consent from participants and protecting confidentiality through anonymization. No personally identifiable information or specific geographic details are included in the datasets, which limits any potential for identification of individuals or locations. Ethical approvals for data collection are documented within the DHS reports. As this study relied solely on publicly available anonymized data, separate ethical approval or informed consent was not required.

## Results

### Socio-demographic characteristics

The socio-demographic characteristics of under-five children and their mothers were analyzed using data from the Demographic and Health Surveys (DHS) conducted in 22 Sub-Saharan African countries between 2013 and 2024. The study included a weighted sample of 109,818 participants.

Among the children, 13.4% were under six months of age, while 49.0% were between 24 and 59 months. The gender distribution was nearly balanced, with 50.7% of the children being male and 49.3% female. Mothers’ ages were predominantly in the 25–34 age group, accounting for 47.5% of the sample. Regarding the educational attainment of mothers’ partners, 31.5% had no formal education, whereas 40.0% had completed secondary or higher education.

Household wealth, as measured by the wealth index, showed that 22.2% of the participants were in the poorest quintile, while 17.5% were in the richest quintile. In terms of residence, 33.4% of the sample lived in urban areas, compared to 66.6% in rural areas. The representation of countries within the dataset varied. Chad and the Democratic Republic of Congo had the largest contributions, representing 9.1% and 9.2% of the sample, respectively, followed by Benin at 7.1%. Conversely, smaller contributions were observed from Lesotho (0.9%), South Africa (1.0%), and Namibia (1.3%). These country-level variations provide a comprehensive regional overview of the study population. Table 1 provides detailed summary statistics for these socio-demographic characteristics.

**Table 1:** Socio-demographic characteristics of under five children and mothers in 22 Sub-Saharan African countries using 2013–2024 DHS (*n* = 109, 818)

Variables	Weighted frequency	Percent (%)
Child age in months		
Less than 6	14,677	13.4
6–23	41,362	37.6
24–59	53, 780	49.0
Child sex		
Male	55, 702	50.7
Female	54, 116	49.3
Birth order		
First	19, 632	17.9
Later	90, 186	82.1
Maternal age in years		
15–24	29, 909	27.2
25–34	52, 209	47.5
>=35	27, 700	25.3
Educational status of women		
No formal education	38, 991	35.5
Primary education	35, 603	32.4
Secondary and above education	35, 224	32.1
Educational status of partner		
No formal education	34, 605	31.5
Primary education	31, 310	28.5
Secondary and above education	43, 903	40.0
Partner occupation		
Not working	8, 865	8.1
Working	100, 260	91.9
Wealth index		
Poorest	24, 212	22.2
Poorer	23, 104	21.0
Middle	21, 777	19.8
Richer	21, 464	19.5
Richest	19, 262	17.5
Place of residence		
Urban	36, 721	33.4
Rural	73, 097	66.6
Sub-Saharan African countries/years		
Angola 2015-16	4892	4.5
Benin 2017-18	7, 742	7.1
Burundi 2016-17	7, 812	7.1
Burkina Faso 2021	5, 767	5.3
Chad 2014-15	10, 020	9.1
Cote d’Ivoire 2021	4, 228	3.9
DR.Congo 2013-14	10, 074	9.2
Gabon 2019-21	2, 196	2.0
Gambia 2019-20	4, 207	3.8
Ghana 2022	3, 898	3.6
Kenya 2022	7, 218	6.6
Lesotho 2023-24	986	0.9
Madagascar 2021	6, 990	6.4
Mozambique 2022	3, 541	3.2
Namibia 2013	1, 477	1.3
Rwanda 2019-20	5, 047	4.6
Senegal 2023	3, 289	3.0
Sierra Leone 2019	5, 160	4.7
South Africa 2016	1, 114	1.0
Tanzania 2022	4, 778	4.4
Zambia 2018	5, 253	4.8
Zimbabwe 2018	4, 130	3.8

### Child survival across socio-demographic and healthcare factors

**Fig. 1:**
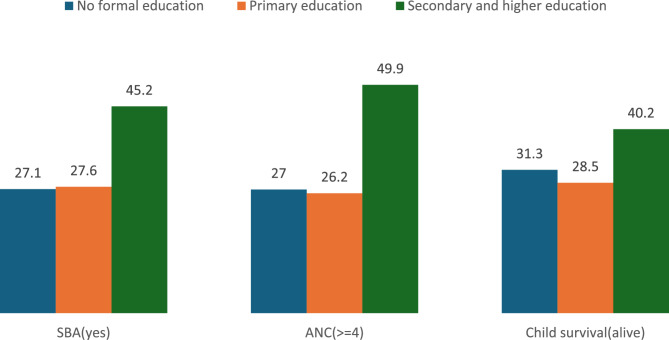
Child and maternal health outcomes with partner education in 22 SSA countries, from 2013–2024 DHS data (*n* = 109, 818)

The overall prevalence of child survival in this study was 96.5% (95% CI: 96.4–96.6%), while 3.5% of children had died. Child survival varied by partner education, with a slightly higher prevalence observed among children of partners with secondary or higher education (96.9%) compared to those with no formal education (96.0%) (Fig. 1).

Age-related differences were also evident, as younger children (under six months and 6–23 months) had a higher survival rate (96.9%) compared to older children aged 24–59 months (96.1%). Female children exhibited better survival rates (96.9%) compared to male children (96.1%). Maternal age was another important factor, with the highest child survival (97.0%) observed among children of mothers aged 25–34 years, while survival was lower for children of mothers aged ≥ 35 years (95.6%).

**Fig. 2:**
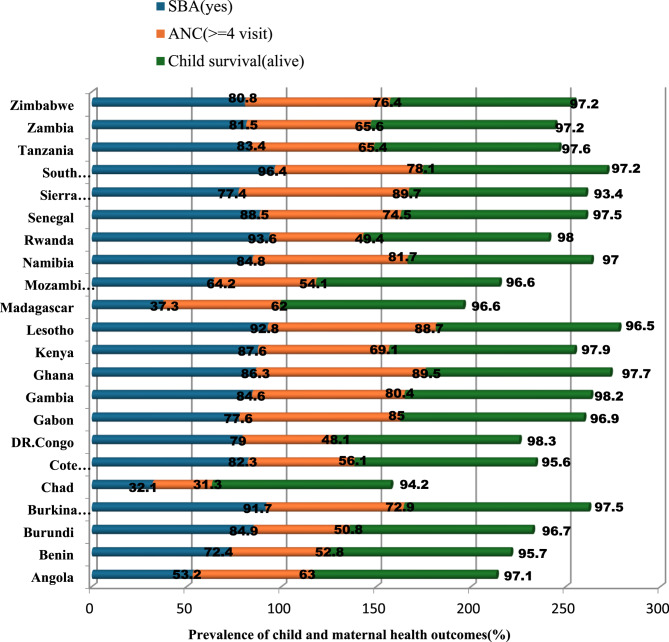
Prevalence of child and maternal health outcomes in 22 SSA countries, from 2013–2024 DHS data (*n* = 109, 818)

Healthcare utilization significantly influenced survival. Children whose mothers attended at least four antenatal care (ANC) visits had the highest survival rates (96.9%), whereas those whose mothers had no ANC visits had the lowest survival rates (94.0%). Residential and regional differences were also noted. Children in urban areas had higher survival rates (96.9%) compared to those in rural areas (96.3%). Regionally, survival was highest in Southern Sub-Saharan Africa (97.3%) and lowest in Central Sub-Saharan Africa (95.6%) (Fig. 2). Detailed data on these variations are presented in Table 2.

**Table 2:** Under five children survival in Sub-Saharan Africa from 2013–2024 DHS (*n* = 109, 818)

Variables	Child Survival		COR with 95% CI	AOR with 95% CI
	Deceased *n* (%)	Alive *n* (%)		
Educational status of partner				
No formal education	1399(4.0)	33,207(96.0)	1	1
Primary education	1082(3.5)	30,227(96.5)	1.12(0.95, 1.32)	0.94(0.84, 1.05)
Secondary and above education	1370(3.1)	42,533(96.9)	1.27(1.04, 1.55)*	0.95(0.84, 1.07)
Child age in months				
Less than 6	456(3.1)	14,220(96.9)	1	1
6–23	1282(3.1)	40,080(96.9)	0.99(0.86, 1.14)	1.01(0.88, 1.14)
24–59	2113(3.9)	51,667(96.1)	0.77(0.62, 0.95)*	0.89(0.78, 1.01)
Child sex				
Male	2151(3.9)	53,550(96.1)	1	1
Female	1700(3.1)	52,417(96.9)	1.20(1.13, 1.28)**	1.25(1.15, 1.35)**
Birth order				
First	677(3.5)	18,955(96.5)	1	1
Later	3174(3.5)	87,012(96.5)	1.0(0.91, 1.11)	1.15(1.04, 1.25)*
Maternal age in years				
15–24	1056(3.5)	28,853(96.5)	1	1
25–34	1587(3.0)	50,621(97.0)	1.21(1.09, 1.35)**	1.11(0.99, 1.22)*
>=35	1207(4.4)	26,493(95.6)	0.84(0.77, 0.93)**	0.78(0.70, 0.88)**
Educational status of women				
No formal education	1628(4.2)	37,363(95.8)	1	1
Primary education	1265(3.6)	34,338(96.4)	1.11(0.92, 1.34)	0.99(0.89, 1.11)
Secondary and above education	959(2.7)	34,266(97.3)	1.49(1.22, 1.81)	1.22(1.06, 1.39)
Partner occupation				
Not working	284(3.2)	8582(96.8)	1	1
Working	3534(3.5)	96,726(96.5)	0.96(0.83, 1.11)	1.03(0.90, 1.17)
Wealth index				
Poorest	921(3.8)	23,291(96.2)	1	1
Poorer	896(3.9)	22,208(96.1)	1.01(0.89, 1.14)	0.99(0.86, 1.13)
Middle	783(3.6)	20,994(96.4)	1.03(0.90, 1.18)	0.98(0.84, 1.15)
Richer	711(3.3)	20,753(96.7)	1.19(1.04, 1.36)	1.06(0.89, 1.27)
Richest	541(2.8)	18,721(97.2)	1.32(1.06, 1.64)	1.05(0.86, 1.29)
ANC utilization				
None	572(6.0)	8973(94.0)	1	1
1–3 visits	1201(3.7)	31,168(96.3)	1.65(1.39, 1.97)**	1.43(1.18, 1.73)**
>=4 visits	2078(3.1)	65,826(96.9)	1.95(1.60, 2.37)**	1.67(1.44, 1.93)**
Skill birth attendant				
No	1243(4.3)	27,503(95.7)	1	1
Yes	2608(3.2)	78,464(96.8)	1.33(1.17, 1.51)**	1.04(0.89, 1.22)
Place of residence				
Urban	1134(3.1)	35,587(96.9)	1	1
Rural	2717(3.7)	70,380(96.3)	0.84(0.77, 0.92)**	0.96(0.84, 1.09)
Regions of SSA countries				
Eastern	1267(2.8)	43,502(97.2)	1.07(0.89, 1.28)	1.02(0.78, 1.33)
Western	1287(3.8)	33,003(96.3)	0.78(0.57, 1.06)	0.72(0.55, 0.95)*
Central	1201(4.4)	25,981(95.6)	0.62(0.49, 0.79)**	0.75(0.57, 0.99)*
Southern	96(2.7)	3481(97.3)	1	1

*AOR* Adjusted Odds Ratio, *SSA* Sub-Saharan Africa

^*^p-value< 0.05

^**^p-value < 0.001

### Relationship between partner education and child survival

Logistic regression analysis was employed to assess the association between partner education and child survival, while adjusting for potential confounders. In the final adjusted model, partner education did not show a statistically significant relationship with child survival.

Among other covariates, maternal education emerged as a significant predictor of child survival. Mothers with secondary or higher education had 22% higher odds of child survival compared to those with no formal education (AOR: 1.22; 95% CI: 1.06–1.39). Child sex was another significant factor, with female children having 25% higher odds of survival than males (AOR: 1.25; 95% CI: 1.15–1.35). Birth order also influenced survival, as later-born children demonstrated higher odds of survival compared to first-borns (AOR: 1.15; 95% CI: 1.04–1.25).

Maternal age exhibited mixed effects. Children of mothers aged 25–34 years had slightly higher odds of survival (AOR: 1.11; 95% CI: 0.99–1.22), whereas children of mothers aged ≥ 35 years had significantly lower odds (AOR: 0.78; 95% CI: 0.70–0.88). Healthcare utilization, particularly ANC visits, had a substantial positive impact. Mothers who attended four or more ANC visits had significantly higher odds of child survival (AOR: 1.67; 95% CI: 1.44–1.93) compared to those who had no ANC visits.

Regional disparities were also evident. Children in Western (AOR: 0.72; 95% CI: 0.55–0.95) and Central Sub-Saharan Africa (AOR: 0.75; 95% CI: 0.57–0.99) had lower odds of survival compared to children in Southern Sub-Saharan Africa. Further details on these associations are presented in Table 2.

### Prevalence of skilled birth attendance (SBA)

The overall prevalence of skilled birth attendance (SBA) among mothers in the 22 Sub-Saharan African (SSA) countries studied was 73.8% (95% CI: 73.6–74.1%). The utilization of SBA was strongly influenced by the educational status of partners. Women whose partners had no formal education reported an SBA utilization rate of 63.6%, whereas this increased significantly to 83.5% for women whose partners had attained secondary or higher education (Fig. 1).

Maternal age also influenced SBA utilization, with the highest rate observed among women aged 25–34 years (75.4%), followed by women aged ≥ 35 years (72.9%). Household wealth demonstrated a clear gradient, as SBA utilization increased from 60.5% among women in the poorest quintile to 90.2% among those in the richest quintile. Access to healthcare services, particularly antenatal care (ANC), played a significant role. Women who attended at least four ANC visits had an SBA utilization rate of 82.5%, compared to only 21.9% for those who had no ANC visits.

Urban residence was associated with higher SBA utilization, with 86.7% of urban women using SBA compared to 67.4% of rural women. Regional variations were evident, with the Southern SSA region reporting the highest SBA utilization (90.6%), followed by the Western (82.2%) and Eastern regions (76.3%) (Fig. 2). Additional details are provided in Table 3.

**Table 3:** Skilled birth attendance among mothers in Sub-Saharan Africa from 2013–2024 DHS (*n* = 109, 818)

Variables	Skilled birth attendance		COR with 95% CI
	No *n* (%)	Yes *n* (%)	
Educational status of partner			
No formal education	12,599(36.4)	22,007(63.6)	1
Primary education	8906(28.5)	22,403(71.6)	1.53(0.77, 3.07)
Secondary and above education	7241(16.5)	36,662(83.5)	2.95(1.58, 5.53)
Maternal age in years			
15–24	8389(28.1)	21,520(71.9)	1
25–34	12,859(24.6)	39,350(75.4)	1.13(1.09, 1.27)*
>=35	7498(27.1)	20,202(72.9)	1.01(0.86, 1.17)
Educational status of women			
No formal education	14,480(37.1)	24,511(62.9)	1
Primary education	9734(27.3)	25,869(72.7)	1.69(0.92, 3.09)
Secondary and above education	4532(12.9)	30,692(87.1)	4.21(2.12, 8.39)**
Partner occupation			
Not working	2315(26.1)	6551(73.9)	1
Working	26,222(26.1)	74,038(73.9)	1.04(0.63, 1.71)
Wealth index			
Poorest	9573(39.5)	14,639(60.5)	1
Poorer	7393(32.0)	15,711(68.0)	1.40(1.14, 1.72)*
Middle	5807(29.7)	15,970(73.3)	1.84(1.28, 2.64)*
Richer	4086(19.0)	17,378(81.0)	2.61(1.36, 4.98)*
Richest	1887(9.8)	17,375(90.2)	5.57(3.57, 8.69)**
ANC utilization			
None	7459(78.1)	2087(21.9)	1
1–3 visits	9390(29.0)	22,979(71.0)	8.68(5.58, 14.81)**
>=4 visits	11,897(17.5)	56,007(82.5)	9.92(7.22, 18.05)**
Exposure to mass media			
No	16,711(38.8)	26,312(61.2)	1
Yes	12,035(18.0)	54,760(82.0)	2.89(2.73, 3.06)**
Place of residence			
Urban	4898(13.3)	31,822(86.7)	1
Rural	23,848(32.6)	49,250(67.4)	0.33(0.24, 0.44)**
Regions of SSA countries			
Eastern	10,604(23.7)	34,165(76.3)	0.39(0.14, 1.04)
Western	6097(17.8)	28,193(82.2)	0.54(0.25, 1.15)
Central	11,709(43.1)	15,474(56.9)	0.14(0.04, 0.45)*
Southern	336(9.4)	3241(90.6)	1

### Relationship between paternal education and skilled birth attendance utilization

The interclass correlation in the empty model indicated that 23.1% of the variability in the prevalence of skilled birth attendance among the target women was attributable to differences across countries. In Model II, which included individual-level determinants, the variability among clusters decreased to 17.1%. However, this variability increased to 20.8% in Model III, which incorporated contextual-level determinants, and then returned to 17.1% in Model IV, the final model combining both individual and contextual-level determinants. The proportional change in variance was 31.1% in Model II, 12.4% in Model III, and 31.0% in Model IV. This demonstrates that the inclusion of individual and contextual-level determinants significantly explained the variance in skilled birth attendance, with the final model (Model IV) providing the most comprehensive explanation of the observed variability.

The fixed and random effects analyses examining the association between paternal education and skilled birth attendance (SBA) in Sub-Saharan Africa, with a focus on Model IV, which incorporates both individual-level and community-level variables. Model IV provides the most comprehensive analysis, offering Adjusted Odds Ratios (AORs) that reflect the independent effect of paternal education on SBA while controlling for other factors. The findings reveal that paternal education significantly influences SBA. Women whose partners have primary education are 1.29 times more likely to have skilled birth attendance compared to those with partners who have no formal education (AOR = 1.29, 95% CI: 1.23, 1.35). This likelihood increases further for women whose partners have secondary or higher education, with an AOR of 1.60 (95% CI: 1.52, 1.67), highlighting the strong positive correlation between higher paternal education and improved SBA.

Maternal education, household wealth, and antenatal care (ANC) utilization significantly influence skilled birth attendance (SBA). Women with primary education were 1.32 times more likely to have SBA (AOR = 1.32, 95% CI: 1.26, 1.37), and those with secondary or higher education were 1.79 times more likely (AOR = 1.79, 95% CI: 1.70, 1.89). Similarly, women from the richest households had 2.41 times greater odds of SBA (AOR = 2.41, 95% CI: 2.25, 2.58). ANC visits showed a strong association, with 1–3 visits increasing the likelihood by 5.72 times (AOR = 5.72, 95% CI: 5.39, 6.06) and 4 + visits by 8.25 times (AOR = 8.25, 95% CI: 7.79, 8.74).”

Geographical location significantly impacts skilled birth attendance (SBA). Rural women were less likely to have SBA than urban women (AOR = 0.66, 95% CI: 0.64, 0.69). Regional disparities were also observed: women in Western and Southern Africa had higher SBA rates compared to Eastern Africa (AOR = 1.36 and 1.31, respectively), while Central Africa had lower rates (AOR = 0.36, 95% CI: 0.35, 0.38) (Table 4).

**Table 4:** Fixed and random effect analyses of the association between paternal education and skilled birth attendance

Variables	Model I (null model)		Model II		Model III	Model IV
			Individual-level variables		Community-level variables	Individual and community-level variables
Educational status of partner						
No formal education			1			1
Primary education			1.10(1.06, 1.15)**			1.29(1.23, 1.35)**
Secondary and above education			1.17(1.12, 1.22)**			1.60(1.52, 1.67)**
Maternal age in years						
15–24			1			1
25–34			1.05(1.01, 1.11)*			1.05(1.01, 1.08)*
>=35			1.06(1.01, 1.10)**			1.06(1.01, 1.10)*
Educational status of women						
No formal education			1			1
Primary education			1.22(1.17, 1.27)**			1.32(1.26, 1.37)**
Secondary and above education			1.84(1.75, 1.94)**			1.79(1.70, 1.89)**
Partner occupation						
Not working						
Working						
Wealth index						
Poorest			1			1
Poorer			1.19(1.14, 1.24)			1.24(1.19, 1.29)**
Middle			1.42(1.05, 1.13)			1.47(1.40, 1.54)**
Richer			1.86(1.77, 1.95)**			1.84(1.75, 1.94)**
Richest			2.73(2.56, 2.90)**			2.41(2.25, 2.58)**
ANC utilization						
None			1			1
1–3 visits			7.71(7.29, 2.24)**			5.72 (5.39, 6.06)**
>=4 visits			12.5(11.8, 13.21)**			8.25(7.79, 8.74)
Exposure to mass media					1	1
No			1		0.47(0.46, 0.48)**	1.22(1.18, 1.26)**
Yes			1.55(1.50, 1.60)**			
Place of residence						1
Urban					1	1
Rural					0.30(0.29, 0.31)*	0.66(0.64, 0.69)**
Regions of SSA countries						
Eastern					1	1
Western					0.95(0.91, 0.99)	1.36(1.30, 1.42)**
Central					0.23(0.22, 0.24)	0.36(0.35, 0.38)**
Southern					1.53(1.37, 1.70)	1.31(1.17, 1.47)**
Random Effects						
Community level variance (Se)	0.986(0.207)**		0.679 (0.145)**		0.864 (0.181)**	0.680(0.143)**
ICC % (95% CI)	23.1% (16.6, 31.2%)		17.1% (11.9, 23.9%)		20.8(14.9, 28.4)	17.1(12.0, 23.8)
PCV (%)	Reference		31.1%		12.4%	31.0%
Model selection						
Log-likelihood	−54772.3	−54121.6		−58543.1		−52109.1
AIC	109566.6	108273.1		117098.3		104256.1
BIC	109566.5	108417.6		117156.0		104439.1

*AIC* Akaike's Information Criterion, *BIC* Bayesian Information Criterion, *ICC* Intra-Cluster Correlation, *PCV* Proportional Change in Variance, *SE* Standard Error, *AOR* Adjusted Odds Ratio

^*^p-value< 0.05

^**^p-value <0.001, SSA Sub-Saharan Africa

### Influence of paternal education on ANC utilization

In this study, 61.8% (95% CI: 61.5–62.1%) of mothers across 22 Sub-Saharan African (SSA) countries reported attending four or more antenatal care (ANC) visits, the recommended standard for adequate maternal healthcare. The educational attainment of partners played a significant role in influencing ANC utilization. Among women whose partners had secondary or higher education, 72.5% reported attending four or more ANC visits, compared to 52.9% of those whose partners had no formal education (Fig. 1).

Maternal age also influenced ANC utilization, with women aged 25–34 years having the highest attendance rates (63.3%), followed by women aged ≥ 35 years (60.0%). Household wealth demonstrated a strong gradient, with the proportion of women attending four or more ANC visits rising from 51.5% among the poorest households to 77.6% in the wealthiest households.

Exposure to mass media emerged as a significant factor, as 69.8% of women exposed to mass media attended four or more ANC visits, compared to 49.5% of women without media exposure. Urban residency was another important determinant; 73.3% of urban women attended four or more ANC visits, compared to only 56.1% of rural women.

Regional differences were pronounced. The Southern region had the highest prevalence of women attending four or more ANC visits (82.5%), followed by the Western region (71.8%). Detailed regional and country-level data are presented in Table 5; Fig. 2.

**Table 5:** Antenatal care service utilization among mothers in Sub-Saharan africa, from 2013–2024 DHS data (*n* = 109, 818)

Variables	ANC utilization			COR with 95% CI
	None *n* (%)	1–3 visits *n* (%)	>=4 visits *n* (%)	
Educational status of partner				
No formal education	5452(15.8)	10,840(31.3)	18,313(52.9)	1
Primary education	2386(7.6)	11,159(35.6)	17,765(56.7)	1.36(0.73, 2.54)
Secondary and above education	1708(3.9)	10,369(23.6)	31,826(72.5)	2.56(1.31, 5.0)
Maternal age in years				
15–24	2735(9.1)	8946(29.9)	18,228(60.9)	1
25–34	4220(8.1)	14,936(28.6)	33,052(63.3)	1.08(1.01, 1.17)*
>=35	2591(9.4)	8486(30.6)	16,623(60.0)	0.95(0.86, 1.06)
Educational status of women				
No formal	6289(16.1)	12,637(32.4)	20,065(51.5)	1
Primary	2434(6.8)	12,389(34.8)	20,780(58.4)	1.56(0.89, 2.74)
Secondary and above	823(2.3)	7343(20.9)	27,059(76.8)	3.47(1.96, 6.14)
Partner occupation				
Not working	825(9.2)	2566(29.0)	5475(61.8)	1
Working	8616(8.5)	29,637(29.6)	62,007(61.9)	1.06(0.73, 1.52)
Wealth index				
Poorest	3444(14.2)	8299(34.3)	12,470(51.5)	1
Poorer	2377(10.3)	7499(32.4)	13,229(57.3)	1.25(1.07, 1.45)*
Middle	1777(8.2)	6780(31.1)	13,220(60.7)	1.43(1.09, 1.87)*
Richer	1418(6.6)	6009(28.0)	14,036(65.4)	1.68(1.10, 2.56)*
Richest	531(2.8)	3782(19.6)	14,949(77.6)	2.99(1.98, 4.50)**
Exposure to mass media				
No	6942(16.1)	14,784(34.4)	21,297(49.5)	1
Yes	2604(3.9)	17,584(26.3)	46,607(69.8)	2.58(1.71, 3.89)
Place of residence				
Urban	1424(3.9)	8385(22.8)	26,912(73.3)	1
Rural	8121(11.1)	23,984(32.8)	40,992(56.1)	0.50(0.39, 0.64)*
Regions of SSA countries				
Eastern	2261(5.1)	15,082(33.7)	27,425(61.3)	0.35(0.25, 0.48)**
Western	1412(4.1)	8273(24.1)	24,606(71.8)	0.52(0.27, 1.01)
Central	5728(21.1)	8531(31.4)	12,922(47.5)	0.14(0.06, 0.29)**
Southern	144(4.0)	482(13.5)	2951(82.5)	1

### Does paternal education influences ANC service utilization?

The interclass correlation in the empty model showed that 6.3% of the variability in antenatal care service utilization among the target women was due to differences across countries. In Model II, which included individual-level determinants, the variability among clusters decreased to 3.6%. This variability further dropped to 3.2% in Model III, which incorporated contextual-level determinants, and reached its lowest point at 2.2% in Model IV, the final model combining both individual and contextual-level determinants. The proportional change in variance was 49.8% in Model II, 43.9% in Model III, and 67.4% in Model IV. This indicates that the inclusion of these determinants significantly accounted for the variance in ANC service utilization, with the final model (Model IV) providing the most substantial explanation of the observed variability.

Using a mixed-effects model (Model IV) that accounted for both individual and community factors, we examined the association between paternal education and antenatal care (ANC) utilization in Sub-Saharan Africa. Compared to women whose partners had no formal education, those with partners who completed primary education were 1.38 times more likely to utilize ANC (AOR = 1.38, 95% CI: 1.33, 1.43). This likelihood significantly increased to 1.99 times (AOR = 1.99, 95% CI: 1.91, 2.07) for women whose partners had secondary or higher education, indicating a strong positive correlation between higher paternal education and improved ANC utilization.

Women aged 25–34 are slightly more likely to utilize ANC compared to younger women aged 15–24 (AOR = 1.08 and 1.09, respectively). Maternal education further amplifies this effect, with women who have primary education being 1.57 times more likely to utilize ANC (AOR = 1.57, 95% CI: 1.52, 1.62), and those with secondary or higher education showing an even stronger association (AOR = 2.26, 95% CI: 2.17, 2.36). Household wealth also significantly impacts ANC utilization, as women from the richest households are 1.36 times more likely to use ANC compared to those from the poorest households (AOR = 1.36, 95% CI: 1.29, 1.44). Exposure to mass media further enhances ANC utilization, with women exposed to media being 1.41 times more likely to seek ANC services (AOR = 1.41, 95% CI: 1.37, 1.45).

Geographical factors also contribute to disparities in ANC utilization. Women in rural areas are less likely to utilize ANC compared to those in urban areas (AOR = 0.82, 95% CI: 0.79, 0.85), highlighting the challenges faced by rural populations in accessing healthcare. Regional differences are also evident, with women in Western and Southern Africa being more likely to utilize ANC compared to those in Eastern Africa (AOR = 2.17 and 1.86, respectively), while women in Central Africa are less likely to do so (AOR = 0.40, 95% CI: 0.38, 0.41) (Table 6).

**Table 6:** Fixed and random effect analyses of the association between paternal education and antenatal care utilization

Variables	Model I (null model)	Model II	Model III	Model IV
		Individual-level variables	Community-level variables	Individual and community-level variables
Educational status of partner				
No formal education		1		1
Primary education		1.06(1.02, 1.09)**		1.38(1.33, 1.43)**
Secondary and above education		1.34(1.29, 1.39)**		1.99(1.91, 2.07)**
Maternal age in years				
15–24		1		1
25–34		1.08(1.05, 1.11)**		1.08(1.05, 1.11)**
>=35		1.07(1.04, 1.11)**		1.09(1.05, 1.13)**
Educational status of women				
No formal		1		1
Primary		1.33(1.29, 1.38)**		1.57(1.52, 1.62)**
Secondary and above		2.17(2.08, 2.26)**		2.26(2.17, 2.36)**
Partner occupation				
Not working				
Working				
Wealth index				
Poorest		1		1
Poorer		1.07(1.07, 1.04)		1.11(1.08, 1.15)**
Middle		1.09(1.05, 1.13)		1.14(1.10, 1.19)**
Richer		1.06(1.02, 1.10)**		1.10(1.06, 1.15)**
Richest		1.37(1.30, 1.43)**		1.36(1.29, 1.44)**
Exposure to mass media				
No		1		1
Yes		1.94(1.89, 1.99)**		1.41 (1.37, 1.45)**
Place of residence				
Urban			1	1
Rural			0.47(0.46, 0.48)**	0.82(0.79, 0.85)**
Regions of SSA countries				
Eastern			1	1
Western			1.38(1.34, 1.42)**	2.17(2.09, 2.25)**
Central			0.35(0.34, 0.36)**	0.40(0.38, 0.41)**
Southern			2.44(2.23, 2.67)**	1.86(1.70, 2.04)**
Random Effects				
Community level variance (Se)	0.221(0.046)**	0.111(0.024)**	0.124 (0.027)**	0.072(0.017)**
ICC % (95% CI)	6.3(4.3, 9.2)	3.2(2.2, 4.9)	3.6(2.4, 5.5)	2.2(1.4, 3.3)
PCV (%)	Reference	49.8%	43.9%	67.4%
Model selection				
Log-likelihood	−99173.8	−5661.77	−93994.7	−89909.9
AIC	198351.6	11353.53	188003.4	179855.9
BIC	194351.4	11467.94	188070.8	180029.2

*AIC *Akaike’s Information Criterion, *BIC *Bayesian Information Criterion, *ICC *Intra-Cluster Correlation, *PCV *Proportional Change in Variance, *SE *Standard Error, *AOR *Adjusted Odds Ratio

*p-value < 0.05

**p-value < 0.001, *SSA *Sub-Saharan Africa

## Discussion

This study explored the relationship between paternal education and maternal and child health outcomes across 22 Sub-Saharan African (SSA) countries. The findings reveal a complex interaction between paternal education, maternal healthcare utilization, and child survival, highlighting the indirect yet significant role fathers play in influencing maternal and child health outcomes. To our knowledge, this is among the first multi-country DHS analyses in SSA to establish a clear association between paternal education and maternal healthcare utilization, and to explore its downstream implications for child survival, setting it apart from prior single-country or maternal-focused studies.

The study found that women with partners who attained secondary or higher education were significantly more likely to utilize maternal healthcare services, including four or more antenatal care (ANC) visits and skilled birth attendance (SBA). This aligns with prior research that associates paternal education with improved maternal health behaviors and service utilization [18, 19]. Educated fathers are more likely to be aware of the importance of healthcare, enabling better resource allocation and support for maternal health-seeking behaviors [20]. A study by Fink et al. [21] also highlighted that higher paternal education improves household income and health literacy, which are crucial determinants of healthcare access. Higher paternal education enhances men’s capacity to interpret health information, support timely care-seeking, and allocate household resources for healthcare. Educated fathers may also exhibit greater support for women’s autonomy in health-related decisions, thereby facilitating maternal healthcare engagement.

Paternal education is significantly and positively associated with maternal healthcare utilization. Similar findings have been reported by Jeong et al. [22], who demonstrated that paternal education contributes to improved child growth and developmental outcomes by influencing household decision-making and maternal health practices. Additionally, Meng and Groth [23] found that higher paternal education levels are linked to a reduced risk of adverse birth outcomes, such as low birth weight and preterm delivery, primarily through enhanced access to and utilization of healthcare services.

Regional disparities in healthcare utilization and child survival identified in this study echo findings from earlier research. For instance, Tessema et al. [24] and McKinnon et al. [25] highlighted significant differences in maternal health service coverage across regions in SSA, with Southern Africa often demonstrating better outcomes compared to Central and Eastern regions. These disparities are attributed to differences in healthcare infrastructure, government investment, and sociocultural attitudes toward maternal health.

The role of maternal education as a stronger predictor of health outcomes compared to paternal education has been widely documented. Our findings align with studies such as those by Sabageh et al. [26] and Singh et al. [27], which emphasized that maternal education equips women with the knowledge and autonomy needed to make informed health decisions. However, our study underscores the complementary role of paternal education, which amplifies the benefits of maternal education by fostering a supportive environment for healthcare access.

Child survival, while not directly linked to paternal education in our multivariable models, was strongly associated with maternal healthcare utilization, particularly ANC and SBA. These findings are consistent with global evidence suggesting that adequate ANC visits reduce neonatal mortality by identifying and managing pregnancy complications. Similarly, skilled birth attendance has been shown to significantly reduce the risk of perinatal and neonatal deaths by ensuring safe delivery practices. It is important to note that while ANC and SBA were necessarily analyzed in relation to the most recent birth, child survival analysis was conducted for all children under five years of age. This approach ensures a more comprehensive assessment of mortality risk rather than limiting the analysis to only the youngest child in each household [28, 29].

Comparing these findings with studies outside SSA, the interplay between paternal education and maternal healthcare utilization appears to be a global phenomenon. For example, research in South Asia by Sharma et al. [30] and Mahmoud et al. [31] demonstrated that paternal education enhances women’s access to institutional deliveries and postnatal care. This indicates that paternal education serves as a key determinant of family health outcomes across diverse settings.

Exposure to mass media, as highlighted in our study, emerged as an important factor influencing maternal healthcare utilization. This finding supports previous research by Nyarko et al. [32], who noted that mass media campaigns significantly improve awareness of maternal health services, leading to increased ANC visits and skilled birth deliveries. The combined effects of paternal education and media exposure suggest a synergistic approach to improving health-seeking behaviors.

The observed rural-urban differences in healthcare utilization and child survival align with earlier findings by Mutunga et al. [33] and Arsenault et al. [20]. Rural women in SSA face greater barriers to accessing healthcare, including transportation challenges, fewer healthcare facilities, and sociocultural constraints. However, urban settings are not exempt from barriers. Overcrowding, strained health infrastructure, and provider shortages in densely populated cities can hinder service quality and timeliness, complicating access for low-income urban residents. These disparities underscore the need for targeted interventions to improve healthcare accessibility in rural areas.

Finally, the finding that maternal age and birth order influence child survival aligns with prior research. Older maternal age has been associated with increased risks of complications during pregnancy and delivery, as reported by Wang et al. [34]. Similarly, first-born children often face higher risks of mortality due to inexperienced maternal caregiving, as documented by Pradhan et al. [35]. These findings emphasize the interplay of biological, behavioral, and structural factors in determining child survival.

Our findings align with global trends highlighting the significance of parental education in improving maternal and child health outcomes. However, the relatively modest impact of paternal education on child survival in SSA compared to other regions may reflect the unique sociocultural dynamics of the region. Studies by Cometto et al. [36] and Tessema et al. [24] have noted that traditional gender roles in SSA often limit fathers’ involvement in direct caregiving, which may attenuate the impact of paternal education on child outcomes. In high-income countries, where gender roles are often less rigid, the impact of paternal education on child health outcomes is more pronounced, as demonstrated by Fink et al. [21] and Balaj et al. [37]. This contrast underscores the need for culturally sensitive strategies that consider the sociocultural context of SSA when designing interventions to enhance paternal involvement in health decisions. Paternal involvement is often shaped by entrenched gender norms that assign caregiving to women and decision-making to men. In patriarchal societies, even educated fathers may defer maternal health decisions to social customs or family elders, limiting the impact of their education. Addressing these cultural barriers is vital for translating paternal education into health action.

### Policy and programmatic implications

The findings emphasize the importance of promoting educational attainment among men as a strategy to improve maternal and child health outcomes in SSA. Policies that encourage male involvement in maternal healthcare programs, such as ANC education sessions, could enhance healthcare utilization and foster shared decision-making within households. Promoting paternal education aligns with gender equality goals by challenging traditional norms and promoting joint responsibility for family health. Educated fathers are more likely to support their partners’ autonomy, contributing to more equitable household dynamics. Practical interventions could include inviting fathers to ANC appointments, creating father-focused health education programs, and introducing paternity leave policies that encourage shared caregiving roles. Community-based initiatives should also address cultural perceptions of masculinity that deter men’s active participation in reproductive health.

Addressing regional disparities in healthcare access is also critical. Efforts to strengthen healthcare infrastructure and remove financial and logistical barriers in underperforming regions, such as Central and Eastern SSA, are essential for achieving equitable health outcomes. Additionally, integrating health and education policies to simultaneously target both social determinants of health could amplify the benefits of health interventions.

### Strengths and limitations

This study has several strengths. The use of nationally representative data from 22 SSA countries ensures comprehensive coverage and generalizability. The large sample size allowed for robust analyses, accounting for the complex survey design and controlling for potential confounders. Furthermore, the study sheds light on the underexplored role of paternal education in maternal and child health, offering a more holistic understanding of health determinants in SSA.

However, certain limitations should be considered. Due to the cross-sectional design, our findings reflect associations, not causation. Longitudinal studies are necessary to establish temporal sequences and validate causal pathways between paternal education and maternal or child health. Longitudinal studies are needed to establish temporal associations and causal pathways. Additionally, the reliance on self-reported data introduces potential recall bias, particularly regarding maternal healthcare utilization. Residual confounding may also be present, as unmeasured factors such as cultural attitudes toward healthcare could influence the observed relationships. Lastly, the study did not explore other paternal factors, such as employment status and health literacy, which may further elucidate the mechanisms by which paternal education influences health outcomes.

## Conclusion

This study highlights a significant association between paternal education and maternal healthcare utilization, which in turn is linked to improved child survival in Sub-Saharan Africa. While maternal education remains a critical determinant of health outcomes, the findings suggest that engaging fathers in health-related interventions could further enhance maternal and child health. Addressing disparities in education and healthcare access, particularly in rural areas and underserved regions, is vital for sustainable improvements in health outcomes. Future research should prioritize longitudinal studies examining how specific paternal factors, such as health literacy, caregiving roles, and involvement in reproductive decision-making, mediate maternal and child health outcomes.

## Data Availability

The dataset used for this study is available in a public, open access repository. The data set can be accessed via https://dhsprogram.com/data/available-datasets.cfm.

## References

[1] World Health Organization. Child mortality and causes of death. WHO https://www.who.int/gho/child_health/mortality/mortality_under_five_text/en. 2024. Accessed 12 Jan 2025.

[2] Sibanda K, Qoko A, Gonese D. Health expenditure, institutional quality, and Under-Five mortality in Sub-Saharan African countries. Int J Environ Res Public Health. 2024;21(3):333. 38541331 10.3390/ijerph21030333 PMC10970304

[3] Simen-Kapeu A, Bogler L, Weber AC, Ntambi J, Zagre NM, Vollmer S, Ekpini RE. Prevalence of diarrhoea, acute respiratory infections, and malaria over time (1995-2017): A regional analysis of 23 countries in West and Central Africa. Journal of global Health. 2021;11:13008. 10.7189/jogh.11.13008 PMC8397278 34484715

[4] Apanga PA, Kumbeni MT. Factors associated with diarrhoea and acute respiratory infection in children under-5 years old in ghana: an analysis of a National cross-sectional survey. BMC Pediatr. 2021;21:1–8. 33581716 10.1186/s12887-021-02546-x PMC7881472

[5] Tekelab T, Chojenta C, Smith R, Loxton D. The impact of antenatal care on neonatal mortality in sub-Saharan africa: A systematic review and meta-analysis. PLoS ONE. 2019;14(9):e0222566. 31518365 10.1371/journal.pone.0222566 PMC6743758

[6] Amouzou A, Ziqi M, Carvajal–Aguirre L, Quinley J. Skilled attendant at birth and newborn survival in Sub–Saharan Africa. Journal of global health. 2017;7(2):020504. 10.7189/jogh.07.020504 PMC5804504 29423181

[7] Yaya S, Bishwajit G, Okonofua F, Uthman OA. Under five mortality patterns and associated maternal risk factors in sub-Saharan africa: a multi-country analysis. PLoS ONE. 2018;13(10):e0205977. 30359408 10.1371/journal.pone.0205977 PMC6201907

[8] Tesfa D, Tiruneh SA, Azanaw MM, Gebremariam AD, Engdaw MT, Kefale B, Abebe B, Dessalegn T. Time to death and its determinants among under-five children in Sub-Saharan Africa using the recent (2010–2018) demographic and health survey data: country-based shared frailty analyses. BMC Pediatr. 2021;21:1–1. 34789187 10.1186/s12887-021-02950-3 PMC8597287

[9] Obse AG, Ataguba JE. Explaining socioeconomic disparities and gaps in the use of antenatal care services in 36 countries in sub-Saharan Africa. Health Policy Plann. 2021;36(5):651–61. 10.1093/heapol/czab036 PMC8173665 33751100

[10] Budu E, Chattu VK, Ahinkorah BO, Seidu AA, Mohammed A, Tetteh JK, Arthur-Holmes F, Adu C, Yaya S. Early age at first childbirth and skilled birth attendance during delivery among young women in sub-Saharan Africa. BMC Pregnancy Childbirth. 2021;21:1–2. 34906105 10.1186/s12884-021-04280-9 PMC8670119

[11] Ekholuenetale M, Wegbom AI, Tudeme G, Onikan A. Household factors associated with infant and under-five mortality in sub-Saharan Africa countries. Int J Child Care Educ Policy. 2020;14:1–5.

[12] Yaya S, Uthman OA, Okonofua F, Bishwajit G. Decomposing the rural-urban gap in the factors of under-five mortality in sub-Saharan africa?? Evidence from 35 countries. BMC Public Health. 2019;19:1–0. 31113395 10.1186/s12889-019-6940-9 PMC6528236

[13] WHO Commission on Social Determinants of Health, World Health Organization. Closing the gap in a generation: health equity through action on the social determinants of health: Commission on Social Determinants of Health final report. World Health Organization; 2008.

[14] Balaj M, York HW, Sripada K, Besnier E, Vonen HD, Aravkin A, Friedman J, Griswold M, Jensen MR, Mohammad T, Mullany EC. Parental education and inequalities in child mortality: a global systematic review and meta-analysis. Lancet. 2021;398(10300):608–20. 34119000 10.1016/S0140-6736(21)00534-1 PMC8363948

[15] Mahmoud OA, Maher SA, El Din Amin GE, Marzouk D. Association of maternal and paternal education and developmental domains in 2–36 months old children in primary care setting in Cairo Egypt. QJM: Int J Med. 2023;116(Supplement1):hcad069–276.

[16] Jeong J, Kim R, Subramanian SV. How consistent are associations between maternal and paternal education and child growth and development outcomes across 39 low-income and middle-income countries? J Epidemiol Community Health. 2018;72(5):434–41. 29439191 10.1136/jech-2017-210102

[17] Meng Y, Groth SW. Fathers count: the impact of paternal risk factors on birth outcomes. Matern Child Health J. 2018;22:401–8. 29218490 10.1007/s10995-017-2407-8 PMC5892832

[18] Caldwell JC. Education as a factor in health development. World Dev. 1990;18(7):1095–110.

[19] Sharma G, et al. The role of men in maternal health outcomes. J Biosoc Sci. 2018;50(2):157–72.

[20] Arsenault C, et al. Healthcare equity in low-income countries. BMJ Global Health. 2019;4:e001425.

[21] Fink G, et al. Long-term effects of education on health. J Dev Econ. 2017;126:57–72.

[22] Jeong J, et al. Maternal and paternal education and child outcomes. J Epidemiol Community Health. 2018;72(5):434–41. 29439191 10.1136/jech-2017-210102

[23] Meng Y, et al. Fathers count: paternal factors in birth outcomes. Matern Child Health J. 2018;22:401–8. 29218490 10.1007/s10995-017-2407-8 PMC5892832

[24] Tessema ZT, et al. Geographic disparities in maternal health services. BMC Health Serv Res. 2022;22:143.35115010

[25] McKinnon B, et al. Regional disparities in healthcare in Africa. Social Sci Med. 2014;119:33–41.

[26] Sabageh AO, et al. Maternal education and child survival in Nigeria. J Public Health Afr. 2020;11:811.

[27] Singh PK, et al. Maternal education and health-seeking behaviors. Public Health. 2021;9:e2874.

[28] Lassi ZS, et al. Interventions to improve maternal health. Cochrane Database Syst Rev. 2020;3:CD012423.

[29] Wang W, et al. Health equity in Africa. Int J Equity Health. 2019;18:203.31881899

[30] Sharma G, et al. South Asian paternal roles in maternal health. J Biosoc Sci. 2017;49(3):421–30.

[31] Mahmoud OA, et al. Maternal and paternal education and child development. QJM. 2023;116(Supplement1):hcad069–276.

[32] Nyarko SH, et al. Education and health access. J Biosoc Sci. 2022;54(4):623–39.

[33] Mutunga CJ, et al. Rural-urban disparities in health services. BMC Health Serv Res. 2020;20:554.32552869

[34] Wang W, et al. Maternal age and child mortality in SSA. Int J Equity Health. 2018;17:45.29665834

[35] Pradhan R, et al. Antenatal care and child survival. BMC Health Serv Res. 2018;18:123.29454347

[36] Cometto G, et al. Strengthening health workforce in Africa. Lancet Glob Health. 2016;4:e495–7.27340004

[37] Balaj M, et al. Parental education and inequalities in child mortality. Lancet. 2021;398(10300):608–20. 34119000 10.1016/S0140-6736(21)00534-1 PMC8363948

